# Comparison of noninvasive three dimensional delayed enhancement MRI of left atrial scar with invasive voltage map by using robust 4D point-to-point registration in patients with atrial fibrillation

**DOI:** 10.1186/1532-429X-18-S1-P210

**Published:** 2016-01-27

**Authors:** Zhen Qian, Xiao Zhou, Wooden Shannon, Naveen Rajpurohit, Sandeep K Goyal, Thomas F Deering, Shizhen Liu, Xiaodong Zhong, Mani Vannan, Venkateshwar Polsani

**Affiliations:** 1 Piedmont Heart Institute, Atlanta, GA USA; 2 Johnson and Johnson, Atlanta, GA USA; 3 MR R&D Collaborations, Siemens Healthcare, Atlanta, GA USA; 4 PLA General Hospital, Beijing, China

## Background

Left Atrial scar imaging using delayed enhancement MRI (DE-MRI) has been proposed as a promising tool to guide ablation strategies in patients with atrial fibrillation (AF). Studies have shown that the scar areas detected by DE-MRI correlate with the low voltage areas on the co-registered electroanatomic voltage map based on surface matching. However, such matching methods did not consider the misalignment of the scar areas: a point-to-point comparison between DE-MRI and voltage map remains problematic. In this study, we proposed a robust 4D (3D of geometry and 1D of scar degree) registration algorithm for the point-to-point comparison of DE-MRI and voltage map. Based on the registered images, we hypothesized that by utilizing complex image information extracted from DE-MRI, we were able to predict the low voltage areas in the co-registered voltage maps.

## Methods

Eleven patients scheduled for ablation for paroxysmal AF were recruited and imaged on a 1.5 T Avanto scanner. DE-MRI was acquired 10 minutes after contrast injection using a 3D inversion-recovery-prepared, respiration-navigated, ECG gated, fast spoiled gradient recalled sequence with fat saturation. Typical acquisition parameters included: TR 500-700 ms, TE 1.34 ms, in-plane resolution 1.1 × 1.1 mm, slice thickness 1.5 mm, FOV 350 mm, and flip angle 10°. At the time of ablation, a detailed pre-ablation bipolar voltage map of the left atrium was created using an electroanatomic mapping system (CARTO; Biosense Webster).

**Figure 1:**
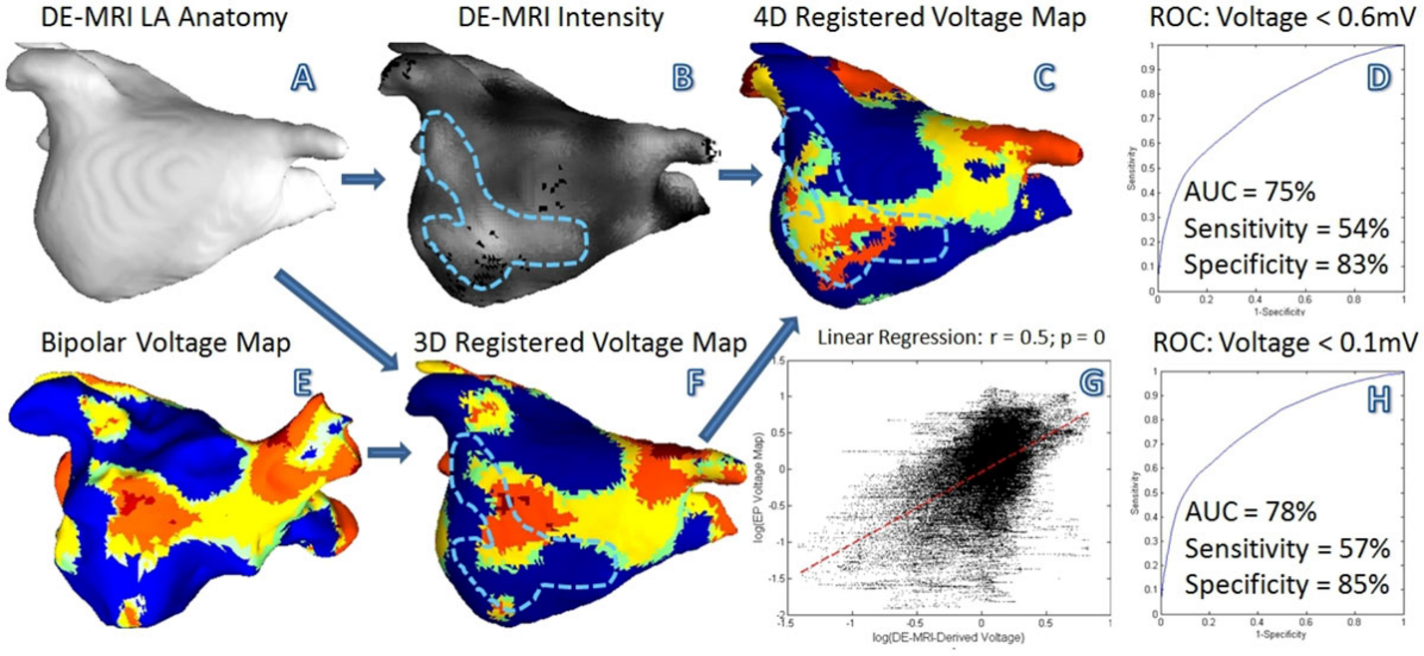
**(A-C;E-G): The flowchart of the 4D point-to-point registration framework**. Note that the high-intensity patch inside the dotted line on the DE-MRI intensity model coincided well with the low voltage area on the 4D registered voltage map, but not on the 3D one. (G): the linear regression results of DE-MRI-derived voltage map and electroanatomic mapping. (D,H): the ROC curves of the DE-MRI-derived voltage map for the detection of low voltage areas of <0.6 mV and <0.1 mV, respectively.

See Figure 1 (A-C;E-G). We adapted a 4D coherent point drift matching framework, which is robust for large deformation and topological variation. Signal intensity in DE-MRI and voltage map were converted to a dimension of scar degree, in which the conversions were iteratively estimated by linear regression and an expectation-maximization strategy. On the point-to-point registered DE-MRI image, a complex intensity profile and the blood pool statistics at each point were extracted. A principal component denoised multivariate regression was performed to estimate the voltage map from DE-MRI. The performance of voltage estimation was measured by linear correlation, and the detection of low voltage area was evaluated using sensitivity, specificity and the area under the ROC curve (AUC).

## Results

See Figure 1 (D,G,H). From the 11 patients, a total of 86854 voltage points were studied. The estimated voltage map by DE-MRI was significantly correlated with the electroanatomic mapping results (r = 0.51, p = 0). DE-MRI detected low voltage areas (< 0.6 mV) with a sensitivity of 54%, a specificity of 83% and an AUC of 75%; DE-MRI detected very low voltage areas (< 0.1 mV) with a sensitivity of 57%, a specificity of 85% and an AUC of 78%.

## Conclusions

Low voltage areas can be identified by DE-MRI using our novel 4D point-to-point registration framework and a more comprehensive analysis of the DE-MRI intensity profile. Such approach may be potentially applicable to clinical ablation guidance but needs further investigation.

